# In Vivo and In Vitro Mechanisms of Equol Synthesis and Key Influencing Factors: A Critical Review

**DOI:** 10.3390/nu17213449

**Published:** 2025-10-31

**Authors:** Tianmeng Zhang, Botao Wang, Chen Wang, Junying Bai, Jingwen Zhou, Jian Chen

**Affiliations:** 1 Key Laboratory of Industrial Biotechnology, Ministry of Education and School of Biotechnology, Jiangnan University, 1800 Lihu Road, Wuxi 214122, China; 2 Science Center for Future Foods, School of Biotechnology, Jiangnan University, Wuxi 214122, China; 3 Bloomage Biotechnology Co., Ltd., Jinan 250101, China; 4 Citrus Research Institute, College of Food Science, Southwest University, Chongqing 400700, China; 5 Key Laboratory of Carbohydrate Chemistry and Biotechnology, Ministry of Education and School of Biotechnology, Jiangnan University, 1800 Lihu Road, Wuxi 214122, China; 6 Jiangsu Provisional Research Center for Bioactive Product Processing Technology, Jiangnan University, 1800 Lihu Road, Wuxi 214122, China

**Keywords:** equol, synthesis, influencing factors, microorganisms

## Abstract

**Background:** Equol exists in two enantiomers of S-equol and R-equol. The results of cell and animal experiments, as well as clinical trials, have supported its protective effects on menopausal symptoms, aging, and cardiovascular diseases, especially S-equol, which is a naturally occurring, non-racemic isomer produced by intestinal bacteria. However, the selective response of host microorganisms to soy isoflavones limits the exploitation of equol-producing bacterial resources. Additionally, factors such as low efficiency, byproduct generation, and environmental pollution hinder the further development and the application of traditional equol synthesis techniques. **Methods:** Therefore, in this review, we aimed to describe the forms and scope of equol, key influencing factors (e.g., hydrogen and dietary factors) of in vivo and in vitro equol synthesis, and potential molecular mechanisms of equol produced by microorganisms. Notably, the traditional synthesis technology has effectively improved the synthesis efficiency of equol (85–96%), but the substrates and microbial species (such as *Escherichia coli*) remain the key influencing factors. **Results:** This review suggests that breakthroughs based on synthetic biology and gene editing technology will support the efficient in vitro synthesis of equol. **Conclusions:** This review serves as a valuable reference for future research.

## 1. Introduction

Numerous studies have demonstrated that the intestinal microbiota mediates the effects of dietary factors on host health and disease [1,2,3], and its core metabolites play a crucial role in its function. Bacteria in the gastrointestinal tract of humans and animals metabolize the soy isoflavone daidzein into S-equol and O-desmethylangolensin (O-DMA) [4,5]. Notably, a variable proportion of human consumers, i.e., from 10 to 60%, were found to have detectable equolplasma levels; the best 50% were designated as equol producers [6,7,8,9]. This difference may be attributed to host dietary habits and the selective results of different microorganisms.

**Figure 1:**
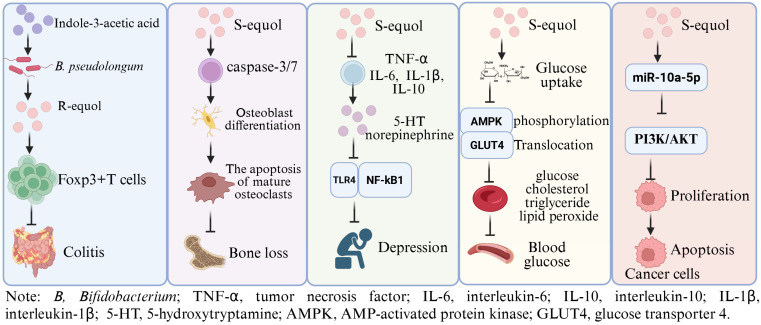
The potential physiological functions of Equol. Indole-3-acetic acid plays a role in alleviating intestinal inflammation by promoting the production of equol by *Bifidobacterium pseudolongum* in mice [19]; S-equol can inhibit osteoclast differentiation and stimulate apoptosis of mature osteoclasts via inducing caspase-3/7 activity in BMCs and RAW 264.7 cells [21]; S-equol can alleviate the depressive-like behavior and neuroinflammation induced by LPS via the TLR4/NF-κB signaling pathway in mice [20]; S-equol can lower the fasting blood sugar level and inhibit the gene expression of liver enzymes related to glucose metabolism in mice, showed a potential anti-diabetic roles [18]; S-equol showed an anti-breast cancer role via up-regulating miR-10a-5p and inhibiting the PI3K/AKT pathway [22]. This Figure was created in BioRender. (2025) https://BioRender.com/fgldagm (accessed on 25 August 2025).

Equol ([7-hydroxy-3-(4′-hydroxyphenyl)-chroman], C_15_H_14_O_3_, molecular weight 242.27) was initially isolated from the urine of a pregnant horse [10], which includes two corresponding isomers: S-equol and R-equol. Notably, epidemiological research has shown that the regular intake of soy isoflavones can reduce the occurrence of estrogen-dependent and aging-related diseases [11,12,13]. The contents of equol and equol-predicting microbial species in urine are closely related to the physiological health of the host, which can be used as a disease marker [14,15]. In particular, S-equol has pharmacokinetic parameters suitable for drug development [16,17] (Supplementary Table S1), endowing it with strong health-supporting functions [18,19,20,21,22] (Figure 1). On this basis, for example, many clinical experimental studies have demonstrated its advantageous roles in preventing age-related bone loss [23], improving skin autofluorescence and visceral fat [24], and these findings suggest its significant potential for application in clinical settings. Presently, relevant data are mainly based on the direct intake of equol or targeted enrichment of equol in the diet. However, synthesis of equol has bacterial and dietary selectivity, which poses challenges for its in vitro synthesis and in vivo regulation. In addition, traditional in vitro synthesis technology is limited by low efficiency, byproduct generation, and environmental pollution, factors that hinder the further development and application of equol.

Over the past decade, although some reviews have summarized the physiological functions, transformation, and metabolism of equol, more research has focused on its physiological effects [25,26]. Unlike these reviews, we have focused on the influence of host physiology (such as microbiome), diet, environment, and other factors on the synthesis of equol, and feasible directions for its biosynthesis. Additionally, we examined the technical and conditional limitations of equol’s applications. We determined that technological breakthroughs based on genomics, synthetic biology, and gene editing may improve the synthesis and application of equol, thereby contributing to the improvement of host genotypes and long-term human microbiota health.

## 2. Search Strategy and Inclusion Criteria

In this review, the PubMed (up to June 2025), Web of Science (1950 to June 2025), and Google Scholar (up to June 2025) databases were utilized to search for the relevant literature. In detail, the following terminology was used for identifying valuable studies: “equol,” “microbes,” “soybean isoflavones,” “synthesis,” “equol-producing bacteria,” “randomized,” “controlled trial,” “mice,” “review,” and “clinical trial”, “in vivo”, and “in vitro”. The included articles mainly focus on the latest literature related to equol synthesis to obtain a more comprehensive set of relevant information. Among them, some articles will be excluded if they are not related to the main theme of this review.

## 3. In Vitro Production of Equol

**Figure 2:**
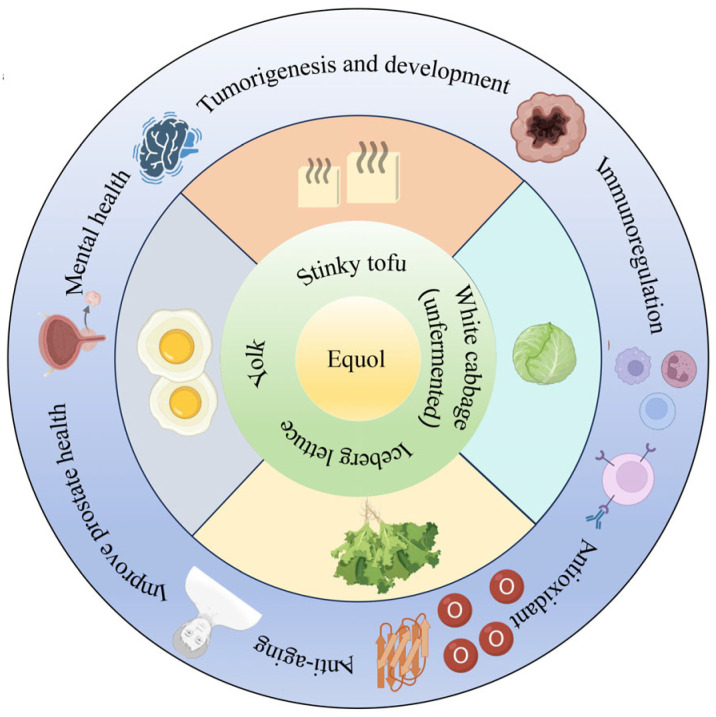
The distribution of equol in human foodstuffs.

Numerous studies have demonstrated that humans have been exposed to equol-rich foods for a considerable period of time in history (Figure 2). Equol exists in fermented soy products, egg yolks [27], cabbage, and lettuce [28,29]; however, the low content in these foods limits equol’s impact on host physiological functions. People who are unable to produce endogenous equol require exogenous equol supplementation to meet their needs. Therefore, the rapid and efficient preparation of equol has become an important research topic.

**Figure 3:**
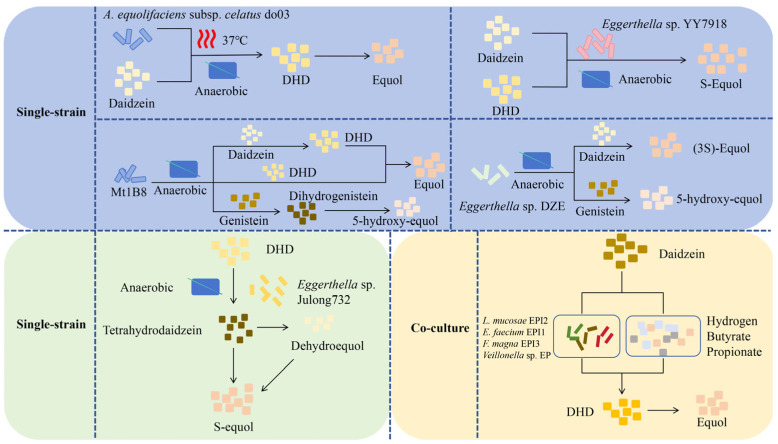
In vitro production method of Equol. (1) Single-strain. Under anaerobic fermentation conditions at 37 °C, *A. equolifaciens* subsp. *celatus* do03 can convert daidzein into equol through dihydrodaidzein (DHD) [33]. *Eggerthella* sp. YY7918 can synthesize S-equol using daidzein and DHD as substrates under anaerobic conditions [32]. The strain Mt1B8 can convert daidzein and genistein into equol and 5-hydroxy-equol, respectively, through DHD and dihydrogenistein (DHG) under anaerobic conditions [36]. Under anaerobic conditions, *Eggerthella* sp. DZE can convert daidzein and genistein into (3S)-equol and 5-hydroxy-equol, respectively [37]. *Eggerthella* sp. Julong732 can convert DHD into S-equol under anaerobic conditions [38]. (2) Co-culture microorganisms. A mixed culture containing four strains (*L. mucosae* EPI2, *E. faecium* EPI1, *F. magna* EPI3, *Veillonella* sp. EP) can convert daidzein into equol through the DHD pathway. Moreover, the colonic fermentation products of carbohydrates such as hydrogen, butyrate, and propionate can stimulate the production of equol [34]. DHD, dihydrodaidzein; *L*, *Lactobacillus*; *E*, *Enterococcus*; *F*, *Finegoldia*; *A*, *Adlercreutzia*.

Intestinal bacteria in humans and mice play a key role in producing equol. The majority of reported soy isoflavone-transforming strains are strictly anaerobic bacteria, including genera such as *Asaccharobacter* [30], *Adlercreutzia* [31], and *Eggerthella* [32], indicating that strains with the same transforming function have a similar taxonomic status. There are two main ways for microorganisms to produce equol (Figure 3): single-strain and co-culturing microorganisms. Minamida et al. (2006) first isolated a Gram-positive bacillus do03 strain capable of converting daidzein to equol from the cecal contents of rats [33]. They found that the rates of conversion of daidzein to equol increased by 4.7 and 4.5 times, respectively, when butyric acid and arginine were added. Compared with murine equol-producing bacteria, equol-producing bacteria isolated from human feces exhibit a higher quantity and diversity [34,35], which may be attributed to the selection of complex dietary factors within the human body. Notably, the metabolic level of equol depends on various factors, including nutritional habits, which influence the composition of the gut microbiota. This indicates that the synergistic effect of multiple equol-producing bacteria can enhance their metabolic efficiency; that is, the ability of an individual to produce equol may also be affected by co-culture with equol-producing bacteria, which provides a basis for further analysis of the differences in equol production among different individuals.

Notably, some microorganisms can only synthesize equol under mixed-culture conditions, but cannot achieve this process under single-culture conditions [34]. Similarly, when different species of microorganisms are compounded in specific proportions, the yield of equol is significantly higher than that of an isolated culture [39]. Although the interactions among these microorganisms and the key factors influencing the generation of equol remain unclear, these findings suggest the possibility of microbial compounding systems promoting the expansion of equol production, as well as the importance of nutrient elements in equol production.

### 3.1. Key Factors Affecting Equol Synthesis

External factors, such as dietary formulas and anaerobic conditions [34], are important factors driving the bioavailability of equol. Daidzein is a crucial nutritional component for the formation of equol [26]. The intestinal flora converts daidzein into equol. After entering the liver, equol can be further metabolized into sulfates or glucuronide conjugates [40,41]. Its metabolites can be excreted into the intestine via bile, and some of them are discharged through urine. However, isoflavones are not the only nutritional elements that are precursors to equol, and some dietary components have demonstrated similar capabilities. For example, indole-3-acetic acid plays a role in alleviating intestinal inflammation by promoting the production of equol by *Bifidobacterium pseudolongum* [19], suggesting that both the equol-generating phenotype and equol generation itself are influenced by dietary habits.

**Figure 4:**
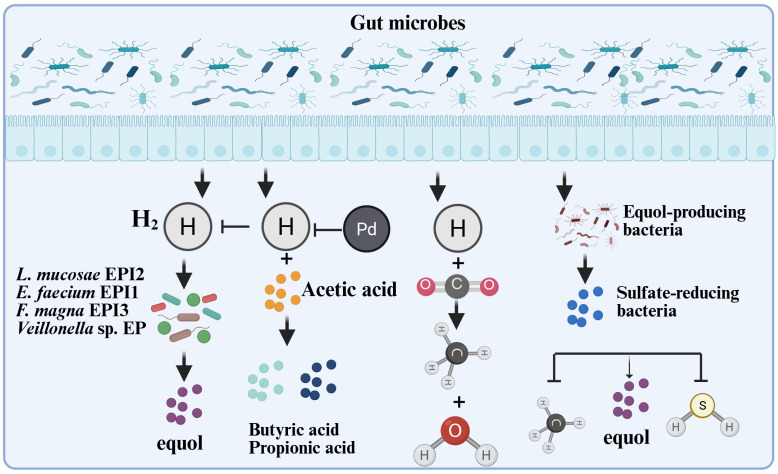
The interaction mechanism between microorganisms, gas, and equol. Hydrogen is an important by-product of anaerobic bacterial fermentation and is partially reused by specific intestinal microorganisms. For instance, methanogens can use H_2_ as an electron donor to reduce CO_2_, methanol, or acetic acid into methane gas [45,46]; the inhibitory effect of acetic acid on the production of equol may be due to the consumption of hydrogen by bacteria that produce propionic acid or butyric acid. H_2_ can stimulate equol production by the mixed culture [34]; the count of equol-producing bacteria is negatively correlated with the count of *Clostridium coccoides-Eubacterium rectale*, and positively correlated with the abundance of sulfate-reducing bacteria [6]; the equol-producing bacterium EPC4 also significantly reduces the generation of methane and hydrogen sulfide in the culture system [44]. This Figure was created in BioRender. (2025) https://BioRender.com/txlg2pd (accessed on 25 August 2025).

Furthermore, in vitro fermentation experiments demonstrated that the colonic fermentation products of indigestible carbohydrates (Figure 4), such as hydrogen, butyric acid, and propionic acid, stimulated equol production in a mixed culture [34]. The existing equol-producing bacteria are mainly anaerobic microorganisms, and most related studies have been conducted under anaerobic conditions. Fecal inoculates of equol producers metabolize daidzein into equol, whereas fecal inoculates of non-equol producers do not produce equol. However, some strains produced O-DMA and dihydrodaidzein, and there were significant differences in the responses of soy isoflavone metabolism in different individuals to the effects of antibiotics [42]. To address this issue, researchers have improved the transformation efficiency and content of equol by optimizing the microbial metabolic environment. Some researchers have established an optimized cascade system by regulating the intensity of gene expression. After optimizing the fermentation conditions, a high S-equol production titer of 3418.5 mg/L was generated [43]. Notably, during the conversion of daidzein into equol, hydrogen gas is consumed by equol-producing bacteria. Under in vitro simulated gastrointestinal conditions, microbial equol production activity weakens the methanogenic and sulfur-producing activities of active hydrogen-consuming bacteria [44], suggesting that hydrogen gas content is one of the main influencing factors affecting equol production. These studies provide a basis for the in vitro synthesis of equol and optimization of related processes.

### 3.2. Biosynthesis of Equol

Chemical synthesis is an important in vitro source of equol. It is mainly based on using isoflavones and chemical reagents to synthesize equol through multi-level chemical reactions. Pelissero et al. (1991) have obtained equol via catalytic hydrogenation of daidzein on palladium/charcoal-EtOH under a hydrogen atmosphere [47]. Although this method showed a highly efficient synthesis efficiency, it has low purity (35%). Based on this, a new chiral stationary phase, Chiralpak^®^ IA, was used for the separation of R- and S-equol. It has good potential in the resolution of the enantiomeric selectivity of equol and can retain the chiral isomers, which is helpful for further evaluation of its metabolites and their enantiomers’ biological effects [48]. Subsequently, many studies have been conducted with the aim of continuously improving chemical synthesis to effectively increase the equol yield [49]. However, the extensive use of chemical reagents inevitably leads to problems such as low purity, the generation of byproducts, and organic reagent residues. The development of efficient, environmentally friendly, and low-cost strategies for the synthesis of equol is currently a research hotspot.

**Figure 5:**
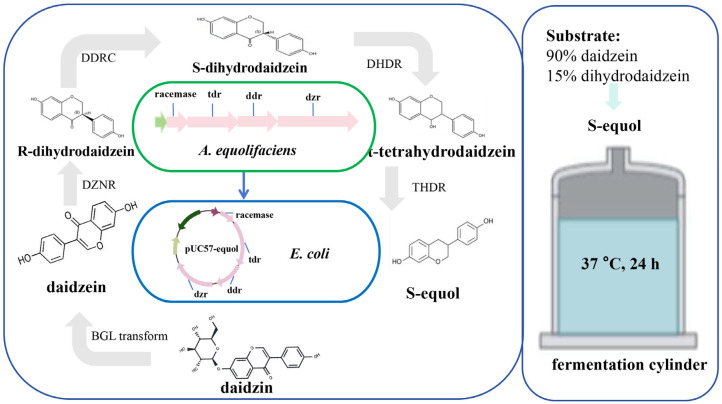
The mechanism of equol synthesis by engineered *E. coli*. The genes (*racemase*, *tdr*, *ddr*, and *dzr*) of four enzymes that are involved in the production of equol in *A. equolifaciens* DSM19450^T^ were synthesized and cloned into a vector (pUC57-Equol) derived from pUC. This vector was introduced into *Escherichia coli* (*E. coli*). The recombinant *E. coli* clone produced equol in a culture medium supplemented with daidzein and dihydrodaidzein [31]. Note: DZNR, Daidzein Reductase; DHDR, Dihydrodaidzein Reductase; THDR, Tetrahydrodaidzein Reductase; DDRC, Dihydrodaidzein Racemase; *A*, *Adlercreutzia*; *E*, *Escherichia*.

In recent years, technologies such as synthetic biology, genomics, and gene editing have shown promise for the rapid and efficient production of equol. Synthetic biology mainly uses biotechnology and intelligent means as the core and organisms as the chassis to process functional substances. By introducing exogenous enzymes or modifying endogenous enzymes, new biosynthetic pathways can be constructed to accumulate target products (Figure 5). Some studies have further emphasized the potential correlation between the *dznr* gene and S-equol content [50,51,52], providing prospects for enhancing in vitro S-equol production. At least four key metabolic enzymes are required to convert daidzein into equol in vitro: DZNR (Daidzein Reductase); DHDR (Dihydrodaidzein Reductase); THDR (Tetrahydrodaidzein Reductase); DDRC (Dihydrodaidzein Racemase). Introducing DZNR, DHDR, DDRC, and THDR enzymes into *E. coli* BL21 (DE3) cells were shown to be an efficient multi-enzyme cascade technique, with a conversion rate of approximately 85.9% [43]. Vazquez et al. (2021) synthesized four genes encoding key enzymes involved in equol production in *Adlercreutzia equolifaciens* DSM19450^T^, cloned them into a vector derived from pUC, and introduced this vector into *E. coli.* Daidzein and dihydrodaidzein were added to the culture medium, and the recombinant *E. coli* produced equol [31]. Notably, in vitro S-equol synthesis efficiency is affected by the external environment (e.g., hydrogen and oxygen). Further application of engineered strains has played a role in the continuous improvement and increased efficiency of equol production (Supplementary Table S2). Wang et al. (2023) constructed recombinant *E. coli* for the biosynthesis of S-equol from soy whey, co-expressing sucrase and α-galactosidase, endowing *E. coli* with the ability to utilize sucrose, raffinose, and trehalose [53]. The optimal strain produced 91.5 mg/L of S-equol in concentrated soybean milk, with a yield of 0.96 mol/mol substrate [53]. The metabolic process of converting soy isoflavones into equol requires strict anaerobic conditions. H_2_ is a metabolic by-product produced by intestinal microorganisms during anaerobic fermentation. Its presence indicates the formation of an anaerobic environment, rather than directly participating in the synthesis of equol. Notably, in some studies, the physiological characteristics and environmental adaptability of equol-producing bacteria have been further modified. Li et al. (2018) constructed and screened transposon mutagenesis libraries to isolate mutant *E. coli* BL21 that is resistant to S-equol, thereby overcoming its inhibitory effect on bacterial growth [54]. The conversion of daidzein into S-equol for efficient production under aerobic conditions provides a convenient method for the in vitro production of equol.

As a new interdisciplinary field, synthetic biology research provides technical support and improvement directions for the in vitro synthesis of equol. However, problems such as the high requirements of synthetic biology technology, a lack of laws and regulations for safety supervision, and unclear protection of intellectual property rights restrict its large-scale promotion in actual production. Furthermore, although oxygen-tolerant bacteria have been developed, these strains grow slowly under aerobic conditions. Screening for oxygen-tolerant strains that can efficiently transform into equol remains the focus of future research.

## 4. Restrictive Factors in the Development of Equol Functions

Notably, there is a difference in the host’s ability to produce equol between humans and animals. Cross-species comparisons between humans and rats indicate that the catalytic efficiency of S-equol production in rats is 210 times that in humans [55], which is mainly attributed to the differences between the intrinsic physiological structures and characteristics of animals and humans. Specific bacteria in human and animal intestines mediate the conversion of isoflavones to equol, and there are significant inter-individual differences in daidzein metabolism. After consuming soybeans or soy extract, approximately 30–50% of people produce equol, and approximately 80–90% produce O-DMA [56]. Direct evidence suggests that only some humans can generate S-equol through microbial metabolism, and this effect is influenced by the sex of the host [57]. Furthermore, among randomized controlled trials involving 110 women, the generation capacity of endogenous equol may have been affected by the vascular function of the host [58]. These results reveal that intrinsic factors such as host sex, intestinal microbiota, and intestinal diseases influence equol generation and transformation efficiency. Furthermore, there are differences in the ability of human and animal models to metabolize equol. Studies have shown significant differences in S-equol metabolic capacity between the liver and intestine in humans, monkeys, dogs, rats, and mice [40]. Additionally, a substantial amount of clinical data provides evidence of a correlation between changes in equol content in plasma and urine and the host’s health status [15,59]. For example, there is a significant difference in daidzein excretion between equol producers and non-equol producers. This partly explains the difference in daidzein bioavailability after soy isoflavone ingestion [60]. In addition, exposure to equol was associated with increased breast cancer risk [61]. There is evidence that equol has a relatively long half-life [17], allowing it to exist in the host for a prolonged period, increasing its opportunities to cross the intestinal and brain barriers [62], thereby affecting the host’s health. Equol mainly exists in plasma in the form of 7-O-glucosidate derivatives [63], which makes it difficult to distinguish between the biologically active forms at the tissue and cellular levels. These factors lead to uncertainties in the functional research and development of equol and in the detection of clinical data. Another challenge is the insufficient understanding of the types and functions of microorganisms involved in equol production. Currently, the vast majority of the reported strains that transform soy isoflavones are strictly anaerobic bacteria and have mainly been studied at the genus level. Moreover, identification of related microorganisms relies mostly on traditional culture methods and microbiological techniques. These factors limit our understanding of the potential value and role of intestinal microorganisms, restricting the further development of related technologies.

## 5. Promotion of Emerging Technologies in Equol-Related Research

The development of models represented by germ-free animals provides the possibility of clarifying the causal relationship between core species and intestinal microbiota equol synthesis. Liang et al. (2020) constructed pseudo Germ-Free mice using broad-spectrum antibiotic interference and established a humanized fecal microbiota mouse model through human fecal microbiota transplantation to simulate the intestinal microbiota of adult human equol producers [64]. The ability to produce equol was partially transferred from human donors to humanized mice [64] and was affected by antibiotics [42]. On this basis, when soybeans were fed to sterile animals and newborn infants lacking a well-developed microbiota, equol could not be detected in the urine [65], whereas when soybeans cultured with fecal microbiota from equol-producing adults were fed to the subjects, they produced S-equol. These results suggest that the yield of equol generated may not depend on the number of equol-producing bacteria but on the physiological characteristics (e.g., antibiotic tolerance) of specific equol-producing bacteria, and that high microbial diversity may increase the yield of equol [66]. Although it has been theorized that the effects of antibiotics on microbial diversity and abundance might be the main reason for the lack of equol production in some organisms, this phenomenon occurred in only 20% of the participants [67]. This result not only provides direct evidence that the gut microbiota drives the synthesis of equol. 

Computer models can intuitively predict and track the modification, degradation, and metabolic processes of equol in the human intestinal tract, thereby providing an accurate reference for potential equol function prediction and development. Some studies have employed in vitro computer simulation methods, including physiologically based pharmacokinetic (PBPK) modeling, to investigate the effects of gut microbiota metabolism on the conversion of daidzein to S-equol and the resulting estrogen activity. This in vitro computer simulation method includes kinetic quantitative analysis of daidzein conversion to microbial metabolites and the establishment of a rat PBPK model that includes intestinal microbiota metabolism [55]. These techniques can be used to explore the role of the gut microbiota in the in vivo effects of equol, predict conditions in the human body, and clarify potential species differences. Goris et al. (2021) [68] analyzed the unified human gastrointestinal protein catalog using known flavonoid modification enzyme sequences as queries and quantified the genes encoding the supposed flavonoid modification enzymes [55]. The enzyme responsible for the conversion of daidzein to equol was more abundant in *A*. *equolifaciens* and an uncharacterized *Eggerthellaceae* species, indicating the hitherto uncharacterized potential of these bacteria to convert daidzein to equol. This finding has substantial implications for further biochemical and microbiological studies on equol transformation. In addition, culturomics combines multiple culture conditions with rapid identification techniques to identify new bacteria, greatly enhancing our understanding of bacteria. On this basis, the application of multi-omics technology, with the continuous advancement of artificial intelligence technologies such as deep learning, will play a more important role in the mining of intestinal microorganisms by improving the efficiency and accuracy of mining. The integration of multi-omics technologies, such as metagenomics, transcriptomics, proteomics, and metabolomics, will provide a more comprehensive understanding of the functions and metabolic pathways of gut microbiota.

## 6. Future Research

The in vitro synthesis of equol remains the main choice for non-equol producers. However, some issues need to be addressed in future research: (1) The synthesis of equol is affected by various factors, such as nutrient sources and oxygen. In the future, by integrating transcriptomics, metabolomics, and molecular biology techniques, the gene regulatory networks and metabolic pathways of aerobic bacteria can be systematically analyzed, laying the foundation for improving their synthetic efficiency. (2) Equol synthesis and metabolic efficiency vary among different bacteria; there may be a synergistic effect between multiple bacteria. The production of equol is not accomplished independently by a single strain, but rather requires the cooperation of multiple microorganisms through metabolic synergy or enzymatic succession. Therefore, it is necessary to conduct a detailed analysis of the signal transduction mechanisms among different strains and reveal the molecular regulatory network for metabolic synergy. (3) Due to the limitations of targeted isolation technology for intestinal microbiota, a large proportion of microorganisms have not been obtained in solid strains. It is necessary to clarify the complex interaction mechanisms among diet, equol, and intestinal microbiota. (4) Considering that there are significant differences in the ability of individuals to metabolize soy isoflavones into equol, it is necessary to identify whether individuals are equol metabolizers, so as to develop more effective nutritional intervention strategies for specific individuals.

## Data Availability

Not applicable. Figures were created with biorender.com.

## Supplementary Materials

The following supporting information can be downloaded at: https://www.mdpi.com/article/10.3390/nu17213449/s1, Table S1: The species/sex differences & pharmacokinetics of equol [16,17,69]; Table S2: The quantitative synthesis for in vitro production [46,53,54,70].
