# Supplementary material for: In Vivo and In Vitro Mechanisms of Equol Synthesis and Key Influencing Factors: A Critical Review
Source: Nutrients. 2025 Oct 31;17(21):3449. doi: 10.3390/nu17213449 (PMC12610683; doi:10.3390/nu17213449)
Supplement: Supplementary file 1 [file nutrients-17-03449-s001.zip › nutrients-3882539-supplementary.pdf]

Supplementary Table 1: The species/sex differences & pharmacokinetics of equol

Ref.	Model	Intervention methods	Sex	Cmax	t½	Conjugation patterns	Transformation potential
[16]	Rats	Gavage dose: 20, 60, and 160 mg/kg bw	male	66.78, 659.38, 2542.02 ng/mL	3.46 ± 2.52, 6.06 ± 3.06, 20.01 ± 9.58 h	-	This study clarified the administration method, which provided a reference for its application in clinical settings.
			female	392.08, 1661.97, 4879.36 ng/mL	NA*, 6.51 ± 1.08, 12.46 ± 4.64 h		
		Intravenous bolus injection: 10 mg/kg bw	male	8563.25 ng/mL	0.28 ± 0.04 h		
			female	8642.91 ng/mL	16.14 ± 14.55 h		
[17]	Rats	Gavage dose: 200 mg/kg diet/day	female	total equol (conjugated and unconjugated): 8815 ± 2988 nmol/L	total equol: 21.9±8.02 h; unconjugated equol: 9.41±3.93 h; equol monosulfate: 14.4±9.26 h; equol glucuronides: 23.0±2.91	equol monosulfate, equol glucuronides	This article elucidated the changes in the complex of equol, which may play a certain role in the potential health benefits of equol.
[69]	Rats	Oral dose (rats): 0, 25, 125, or 500 mg/kg bw	male	Unconjugated S-equol: 21, 830, 2456 ng/mL; total S-equol: 4798, 32480, 49504 ng/mL	Unconjugated S-equol: 5.44 to 10.9 h	Protein-bond	S-equol has pharmacokinetic parameters suitable for drug development with a low potential for

					uterotropic effects.
Monkeys	Oral dose (rats): 0, 125, 250, or 1000 mg/kg bw	male	Unconjugated S-equol: 15.8 ± 11.9, 41.6 ± 19.0, 358 ± 510 ng/mL; total S-equol: 14076 ± 5550, 34329 ± 8381, 85100 ± 21495 ng/mL	Unconjugated S-equol: 2.60 to 5.09 h; total S-equol: 3.44 to 4.21 h.	

Note: *NA Calculated values were considered as outliers.

Supplementary Table 2: The quantitative synthesis for *in vitro* production.

Ref.	Strain/consortium	Substrates	Production titer	Productive rate	Culture conditions		
					pH	Temperature	Headspace gases
[46]	*E. coli*	Daidzein	3418.5 mg/L	85.9%	7.0–8.0	37 °C	oxygen
[54]	*E. coli*	Daidzin	4.1 mg/L	14.2%	-	37 °C	oxygen
[53]	*E. coli*	Soy whey	91.5 mg/L	96%	7.5	37 °C	oxygen
[70]	*E. coli*	Daidzein	69.8 mg/L	95%	7.0	30 °C	oxygen

Note: *A*, *Adlercreutzia*; *E*, *Escherichia*.
